# A heuristic model for computational prediction of human branch point sequence

**DOI:** 10.1186/s12859-017-1864-9

**Published:** 2017-10-24

**Authors:** Jia Wen, Jue Wang, Qing Zhang, Dianjing Guo

**Affiliations:** School of Life Science, State Key Laboratory of Agrobiotechnology and ShenZhen Research Institute, The Chinese University of Hong Kong, Hong Kong, China

**Keywords:** Heuristic model, Bps, Pre-mRNA splicing, Binding energy, Genome-wide prediction

## Abstract

**Background:**

Pre-mRNA splicing is the removal of introns from precursor mRNAs (pre-mRNAs) and the concurrent ligation of the flanking exons to generate mature mRNA. This process is catalyzed by the spliceosome, where the splicing factor 1 (SF1) specifically recognizes the seven-nucleotide branch point sequence (BPS) and the U2 snRNP later displaces the SF1 and binds to the BPS. In mammals, the degeneracy of BPS motifs together with the lack of a large set of experimentally verified BPSs complicates the task of BPS prediction in silico.

**Results:**

In this paper, we develop a simple and yet efficient heuristic model for human BPS prediction based on a novel scoring scheme, which quantifies the splicing strength of putative BPSs. The candidate BPS is restricted exclusively within a defined BPS search region to avoid the influences of other elements in the intron and therefore the prediction accuracy is improved. Moreover, using two types of relative frequencies for human BPS prediction, we demonstrate our model outperformed other current implementations on experimentally verified human introns.

**Conclusion:**

We propose that the binding energy contributes to the molecular recognition involved in human pre-mRNA splicing. In addition, a genome-wide human BPS prediction is carried out. The characteristics of predicted BPSs are in accordance with experimentally verified human BPSs, and branch site positions relative to the 3’ss and the 5’end of the shortened AGEZ are consistent with the results of published papers. Meanwhile, a webserver for BPS predictor is freely available at http://biocomputer.bio.cuhk.edu.hk/BPS.

**Electronic supplementary material:**

The online version of this article (10.1186/s12859-017-1864-9) contains supplementary material, which is available to authorized users.

## Background

In eukaryotes, introns are removed from the pre-mRNA after transcription and exons are joined together by an event named splicing. Pre-mRNA splicing provides a mechanism to generate multiple mRNA isoforms from a single gene and regulates the gene expression post-transcriptionally. Sometimes multiple transcripts can be produced by “alternative splicing”, which plays important role in the regulation of many physiological processes, such as cell differentiation and development. Defects in pre-mRNA splicing underlie a considerable number of genetic diseases and cancers [1–4].

Splicing is a set of reactions catalyzed by the spliceosome, which consists of U1, U2, U4, U5 and U6 snRNPs, and hundreds of non-snRNP proteins [5]. Splicing proceeds through two sequential trans-esterification reactions: the first forms a lariat intermediate with the 5′-end of the intron linked to the branch site positioned within the BPS, and the second results in a complete intron removal and exon ligation [6]. Through alternative splicing process, the transcription of a gene can generate multiple isoforms by selectively removing different intron sequences [2], and thus contributes significantly to the proteome complexity in metazoan [1, 7]. The importance of splicing is demonstrated by the fact that at least 15% of human genetic diseases are caused by mutations at the splicing sites or at the cis-acting splicing regulatory sites [8–10].

In mammalian spliceosome assembly, the U1 snRNP, the SF1, the 65 kDa subunit of U2AF (U2AF65) and the 35 kDa subunit of U2AF (U2AF35) respectively recognize the 5′-splice site (5’ss), the BPS, the polypyrimidine tract (PPT) and the 3′-splice site (3’ss) to form the early E complex [11, 12]. The SF1 is then replaced by the U2 snRNP through a binding between the conserved GUAGUA hexanucleotide in U2 snRNP and the BPS, forming the A complex. This is usually the key step in defining the ends of the intron to be spliced out and the ends of the exon to be reserved [13, 14]. Some splicing auxiliary proteins that bind to the cis-acting splicing sites also regulate the pre-mRNA splicing process by either disrupting or facilitating spliceosome assembly at the correct splicing sites [4, 15, 16].

The branch point sequence (BPS) in yeast is a nearly invariant sequence of UACUA*A*C with the branch site adenosine (*A*) being the sixth nucleotide. This motif is perfectly complementary to the GUAGUA motif in U2 snRNP. However, the BPSs in human are more degenerative and so far we still lack a large “gold standard” set of BPSs verified from the experiment. This complicates the recognition of BPS based on the sequence alone and makes the computational identification of BPS a rather challenging task [17–19]. Although the BPSs have been successfully predicted in fungal species based on the Hamming distance to the U2 complementary sequence [20], this model was proved to be insufficient in mammals [21]. Recently, Corvelo et al. [21] proposed a Support Vector Machine (SVM) algorithm for BPS prediction by training a set of high-confidence putative BPSs, and achieved by far the best prediction accuracy. However, the construction of putative BPSs involves multiple statistical tests which are rather complex. Moreover, the SVM method is only suitable for predicting BPSs with the “TNA” structure.

With the development of sequencing technology, Taggart et al. [22] conducted the first large-scale mapping of BPSs in human pre-mRNA transcripts. Later, LaSSO was developed to map the location of branch sites on genomic scale [23]. Furthermore, Mercer et al. [24] provided the first map of splicing sites in human genome. Nowadays, even though more human BPS data are becoming available, these mapping results have not been verified by wet lab experiment and some of them are incorrect due to mismatch error, micro-insertion or deletion in the generated cDNA [25, 26]. Therefore, more efficient computational methods for human BPS prediction are still in high demand.

In this paper, we develop a novel scoring scheme to quantify the splicing strength of putative BPSs in a newly defined BPS search region. The conservative property of putative BPS, the binding energy between putative BPS and U2 snRNP, and the nucleotide preference of branch site were integrated in the scheme. Associating with two kinds of relative frequencies, we demonstrate the utility of this model on two sets of experimentally verified human introns. Compared with the SVM model, our method can further improve the prediction accuracy of human BPS. We speculate that the binding energy between the BPS and U2 snRNP may contribute to the molecular recognition involved in pre-mRNA splicing. In addition, a genome-wide human BPS prediction is carried out based on our model. The characteristics of predicted BPSs are in accordance with experimentally verified human BPSs, and branch site positions relative to the 3’ss and the 5’end of the shortened AGEZ are consistent with the results of published papers. A webserver for the BPS predictor is freely available at http://biocomputer.bio.cuhk.edu.hk/BPS.

## Methods

### Datasets

Three sets of human intronic data were used in this research. Additional file 2: Dataset S1 and Additional file 3: Dataset S2 are experimentally verified human introns. The Additional file 2: Dataset S1 proposed by Corvelo et al. [21] contains 42 introns, and the Additional file 3: Dataset S2 contains manually curated 88 introns. In addition, all human reference introns with canonical 5’ss and 3’ss (GT and AG, respectively) and with length > 100 bps were also included in this study, which contains a total of 459,678 human introns.

### The identification of PPT region in an intron

As one of important cis-acting elements directing the intron removal in pre-mRNA splicing, the polypyrimidine tract (PPT) commonly locates in between the BPS and the 3’ss. The PPT not only increases the efficiency of branch site utilization, it also functions in the selection of alternative branch sites and thus the 3′ splicing site recognition [27]. Moreover, the degeneracy of human BPSs suggests that they are likely to be recognized in combination with the PPT and other splicing cis-elements [26]. As described by Corvelo et al. [21], the PPT region can be identified based on the following characteristics, maximizing for length:

1. Both ends of the PPT (3′- and 5′-) must be pyrimidines;
2. No more than two continuous purines are allowed;
3. Each purine segment (length *l* < 3) must be surrounded by at least 4 *l* pyrimidines, and both upstream and downstream pyrimidine segments are of length greater or equal to *l*;
4. T(GT)*n* stretches are allowed;
5. Minimum length of the PPT is of 9, or uridine content is greater or equal to 5.

In addition, when multiple PPT candidates are identified in an intron, the one which is closest to the 3’ss is selected.

### The shortened AGEZ of an intron

BPS and PPT are two consensus elements adjacent to the 3’ss. The region between BPS and the 3’ss marked by the absence of AG dinucleotide is named AG exclusion zone (AGEZ), which is defined as the region from the 3’ss to the first upstream AG, ignoring any AG found in the first 12 nucleotides. Furthermore, additional *L* (=7–12) nucleotides upstream the AGEZ-defining AG dinucleotide are also included [17, 21].

**Fig. 1:**
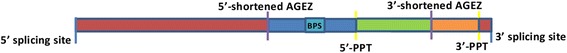
Schematic representation of an intron marked with the shortened AGEZ and the PPT. The shortened AGEZ is in blue and the green. The PPT is in green and orange, which has the highest pyrimidine component starting from the 3′ end of the PPT

The BPS recognition is highly dependent on the presence of the downstream PPT, and a strong correlation between the strength of the PPT and branch site selection is suggested [27]. Based on the characteristics of the PPT suggested by Corvelo et al. [21], a clear pyrimidine-rich signal near the 3′ end of the PPT was observed, and the branch sites usually do not exist in the region. We therefore search the BPS in the AGEZ of an intron excluding the pyrimidine-rich 3′ end of the PPT, and we name it the shortened AGEZ. The shortened AGEZ in an intron is illustrated in Fig. 1.

### A new scoring scheme

Progress has been made using the consensus sequence for BPS prediction [17–19]. However, the low information content of human BPS signals indicates that it is difficult to accurately predict BPS based on the consensus sequence. Hence, we adopt the following strategy to quantify the splicing strength of putative BPSs.

1. Position-specific score

The position-specific scoring matrix (PSSM) was utilized to depict the relative frequencies of each nucleotide at specific position for motif pattern [28]. Based on the PSSM of experimentally verified human BPSs, the position-specific score can be used to quantify the conservative property of each BPS [19, 29].

The position-specific score (S) is calculated as follows:

1 $$ \mathrm{S}={\sum}_{i=1}^7{\mathrm{log}}_2\left({{f_i}_{,x}}_i\right),{x}_i\in \left\{A,C,G,T\right\}, $$

where *f*_*i*,*xi*_ is the frequency of the *i*-th nucleotide in a heptanucleotide *x* at position *i*.

(2) BPS-U2 snRNP binding stability

The base-pairing between the BPS and U2 snRNP is an important step in pre-mRNA splicing, and the BPS-U2 binding stability is considered an important factor for overall splicing efficiency [21]. During the splicing process, a conserved GUAGUA motif within the U2 snRNP can hybridize to the hexanucleotide BPS excluding the branch site. Therefore, the binding stability between putative BPS and U2 snRNP is measured by the binding energy of the hexamer and the GUAGUA motif in U2 snRNP, which can be obtained by RNAcofold in the Vienna RNA package with default parameters [30].

The branch site sometimes cannot be exactly pinpointed by the experiment due to mismatch error, micro-insertion or deletion in the generated cDNA [25], and the reverse transcriptase might skip one or two nucleotides at the branch site [26]. These indicate that the binding stability between putative BPS and U2 snRNP may be affected by the neighboring bases of branch site.

Hence, integrating the conservative property of BPS, the binding energy between the BPS and U2 snRNP, and nucleotide preference of branch site, a series of scoring measures were proposed to quantify the splicing strength of putative BPSs as follows, and the one gives the best result was chosen to predict human BPS:

2 $$ {\mathrm{S}}^{\ast }={\sum}_{i=1}^7{log}_2\left({{f_{i,}}_X}_i\right)-{\sum}_{j=1}^3\left({\sum}_{k=5}^7{P_j}^{\ast } BE{\left(X/{X}_k\right)}^{\ast }{f}_{k,}{{{{}_X}_k}^Q}_j\right)/{\sum}_{j=1}^3{P}_j, $$

where ${f}_{i,{X}_i}$ is the relative frequency of the *i-*th nucleotide in a heptanucleotide *X* at position *i*, *BE*(*X*/*X*_*k*_) is the binding energy between the GUAGUA in U2 snRNP and the heptamer *X* excluding the branch site (*k* = 6) or its neighbors (*k* = 5, 7), and *P*_*j*_, Q_*j*_ ∈ [0, 1]. Specifically, the S* was defined as S in (1) when *P*_*j*_ = 0, *j* = 1, 2, 3.

Based on the definition of Score in (2), 15 scoring measures (score0-score14) are listed (Additional file 1: Table S1). Our model can avoid the complex model training process, and directly quantify all the putative BPSs in the shortened AGEZ of an intron, of which the heptamer with the highest score is predicted as the candidate BPS. For introns containing more than one BPS, we consider a prediction as correct if any one of the BPSs is detected [21].

## Results and discussion

### The shortened AGEZ is efficient for BPS search

**Fig. 2:**
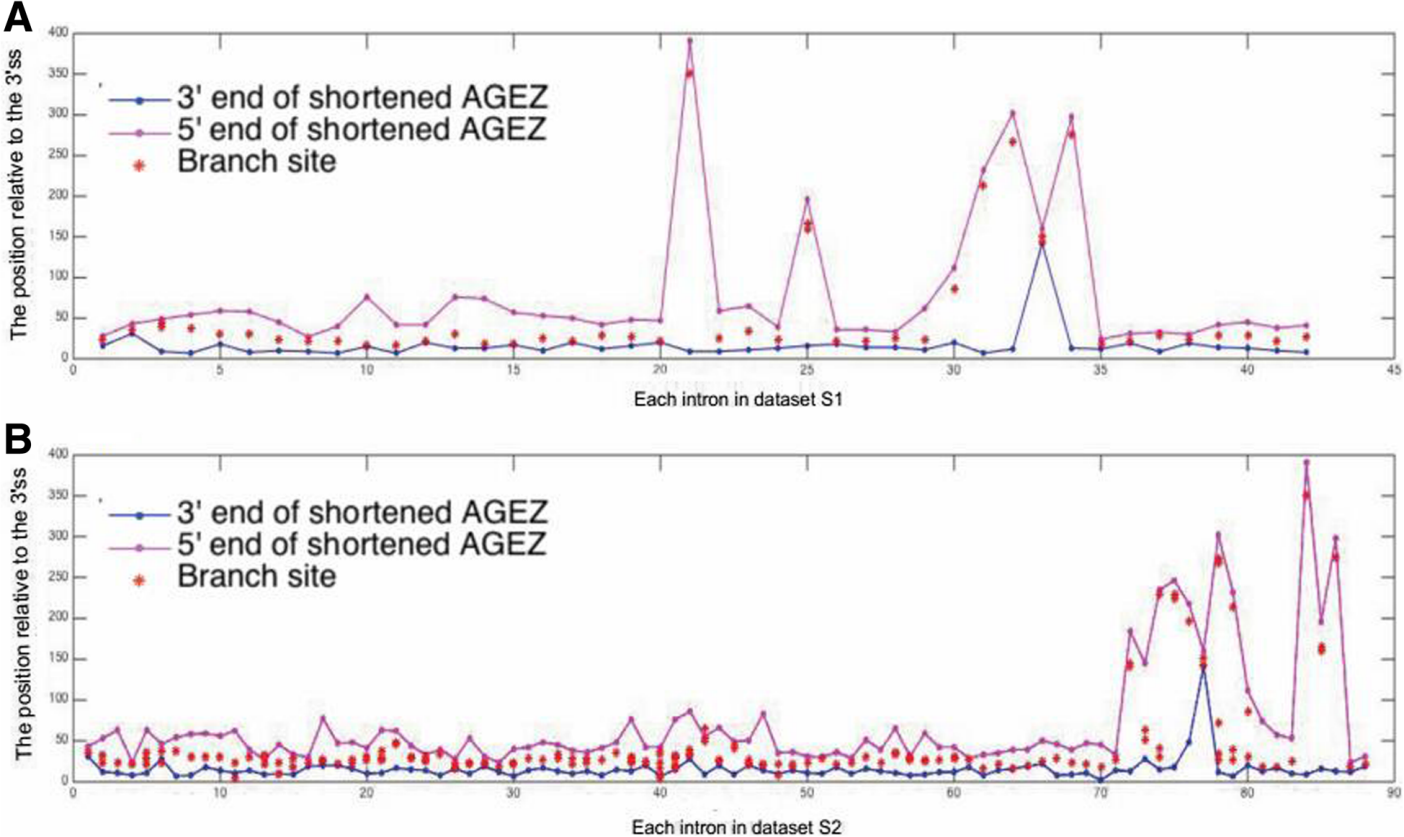
The endpoints (5′- and 3′-) of the shortened AGEZ and corresponding branch sites labelled by their positions relative to the 3’ss for each intron in Additional file 2: Dataset S1 (**a**) and Additional file 3: Dataset S2 (**b**). X-axis: No. of intron in Additional file 2: Dataset S1 (shown in Table S2) and Additional file 3: Dataset S2 (shown in Table S3). Y-axis: Position relative to the 3’ss. Each vertical line represents an intron, in which the dots with different colors depict the 5′/3′ end of the shortened AGEZ (purple/blue) and branch sites (red)

Our newly defined shortened AGEZ constrains the BPS search within a shorter region, and ensures that most branch sites are not missed. To examine if the shortened AGEZ is efficient enough for BPS search, we first marked the shortened AGEZ for each intron in Additional file 2: Dataset S1 and Additional file 3: Dataset S2, respectively. The endpoints of the shortened AGEZ and corresponding branch sites labeled by their positions relative to the 3’ss were then shown in Fig. 2a-b (Additional file 1: Tables S2 and S3). From Fig. 2a-b, all the branch sites are laying in the shortened AGEZ, indicating the shortened AGEZ is efficient for BPS search. Hence, we used the shortened AGEZ to replace the traditional AGEZ for further study [17, 21].

### Characteristics analysis of human BPSs

**Fig. 3:**
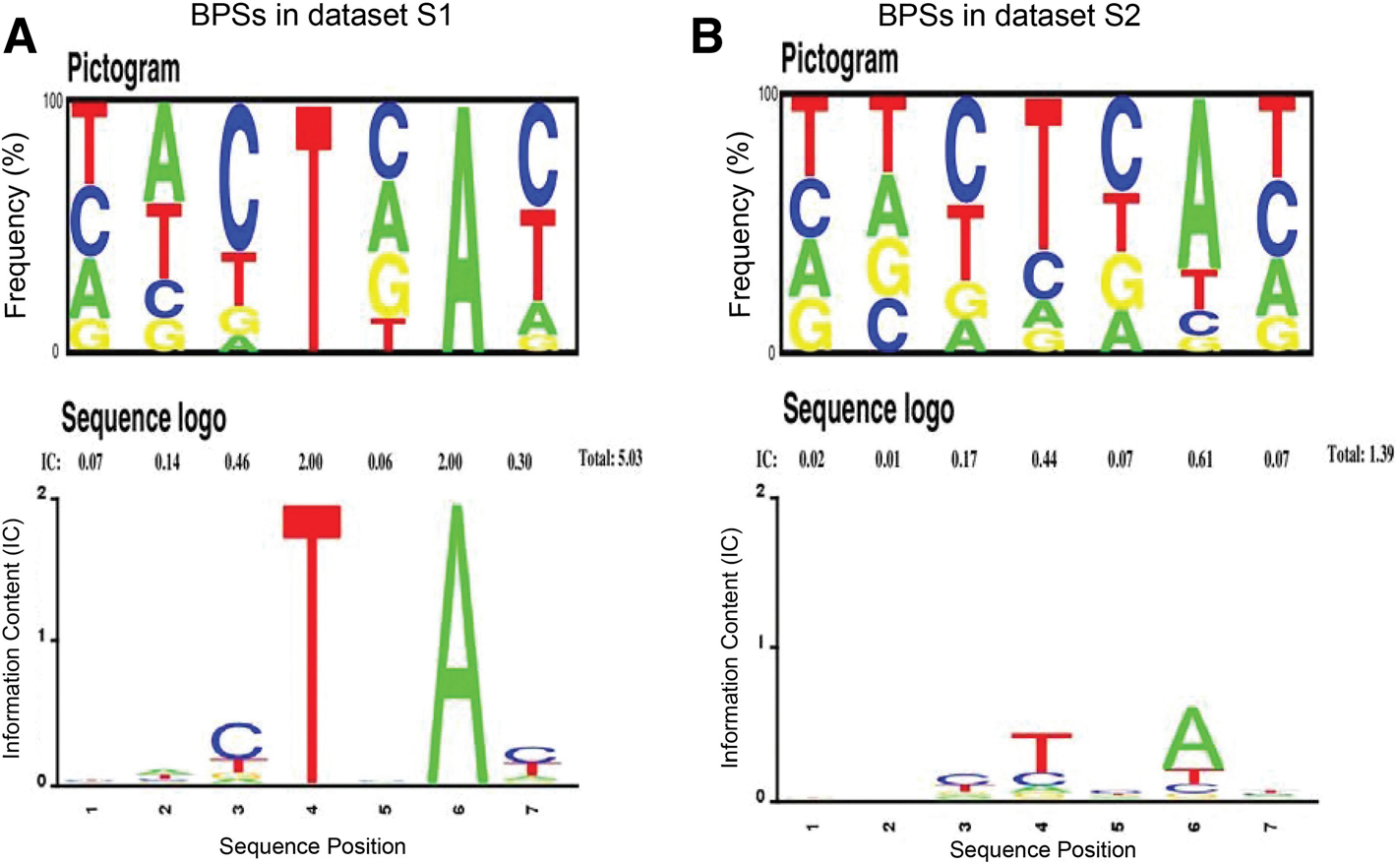
Pictogram and Sequence logo for BPSs in Additional file 2: Dataset S1 (**a**) and Additional file 3: Dataset S2 (**b**). In Pictogram, the height of each letter is proportional to the frequency of nucleotide at the given position; For Sequence logo, the height of letters describes the information content in bits at each position

To analyze the characteristics of human BPSs in Additional file 2: Dataset S1 and Additional file 3: Dataset S2, Pictogram was used to depict relative frequencies of nucleotides at each position [6], and information content (IC) was used to evaluate the conservatism of human BPSs at each specific position [31]. As shown in Fig. 3a-b (Additional file 1: Tables S4 and S5), the human BPSs in Additional file 3: Dataset S2 (total IC = 1.39) are not as conserved as those in Additional file 2: Dataset S1 (total IC = 5.03, with fixed T and A positions of “TN*A*”), and the consensus sequence of human BPS is likely YUN*A*Y [26], where Y is a pyrimidine and N is any nucleotide, rather than CUR*A*Y [32], YNCUR*A*Y [33], YNCUR*A*C [34] or YNYUR*A*Y [35] (R is a purine).

**Fig. 4:**
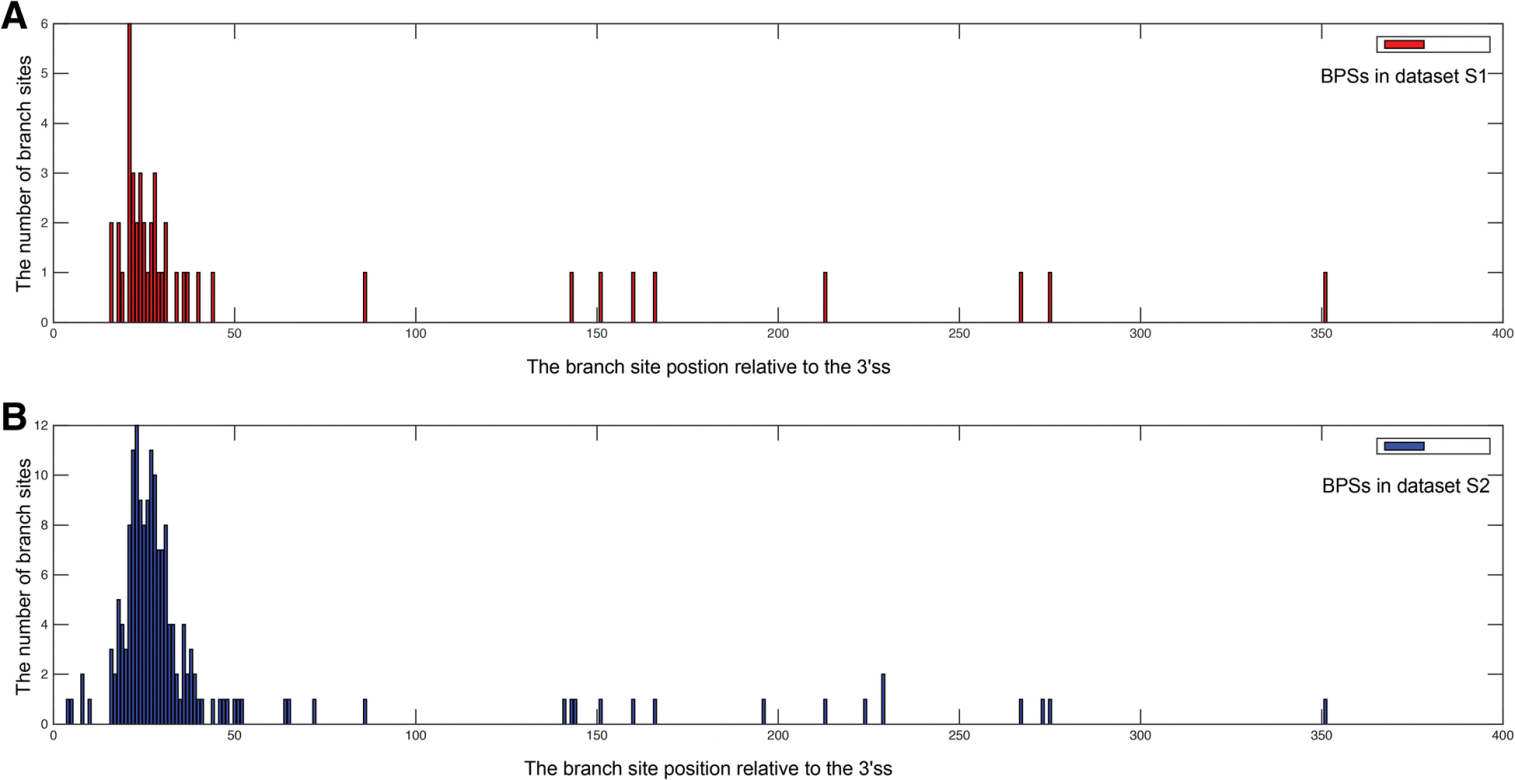
The distributions of BPSs in Additional file 2: Dataset S1 (**a**) and Additional file 3: Dataset S2 (**b**) labeled by their branch site positions relative to the 3’ss

The branch site position relative to the 3’ss was considered an important indicator for BPS prediction. Plass et al. [18] and Schwartz et al. [19] searched over a region of a fixed length (100 nts and 200 nts, respectively) to find candidate BPS. To explore the relative distance between the branch site and the 3’ss for Additional file 2: Dataset S1 and Additional file 3: Dataset S2, the branch site positions relative to the 3’ss were marked in Fig. 4a-b (Additional file 1: Tables S2 and S3). As seen, most branch sites are located within −14 to −45 nts of the 3’ss, and some are even located up to −350 nts of the region. This indicates that it is reasonable to adopt a dynamic search region when different types of human introns are involved.

### The performances of 15 scoring measures in BPS prediction

To demonstrate the utility of our model, two sets of experimentally verified human introns (Additional file 2: Dataset S1 and Additional file 3: Dataset S2) were used for BPS prediction. In addition, two types of relative frequencies (I and II) were utilized: type I contains two frequency matrices shown in Tables S4 and S5 (Additional file 1), which were used to analyze the characteristics of BPSs in Additional file 2: Dataset S1 and Additional file 3: Dataset S2 in the above section, and type II is computed by 252,302 BPSs predicted by sequencing method [24], of which branch sites lie in the shortened AGEZ for all the human introns considered in this study, and the relative frequencies of nucleotides at each position is shown in Table S6 (Additional file 1).

**Fig. 5:**
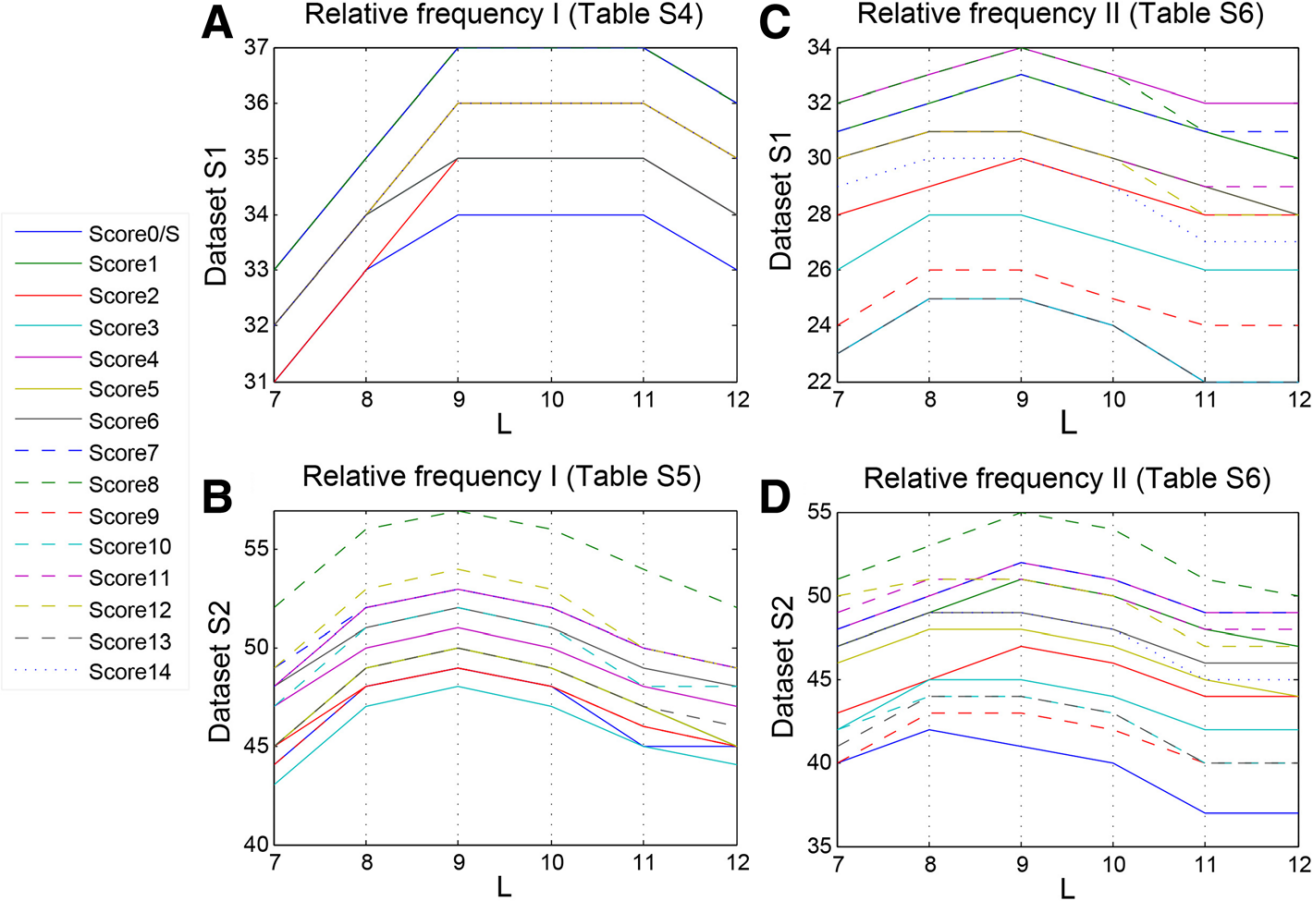
The performances of 15 different scoring measures (Score0-Score14) on Additional file 2: Dataset S1 (**a**, **c**) and Additional file 3: Dataset S2 (**b**, **d**) based on the relative frequencies shown in Additional file 1: Tables S4-S6

Based on the relative frequencies shown in Tables S4-S6 (Additional file 1), 15 different scoring measures (score0-score14) were applied on Additional file 2: Dataset S1 and Additional file 3: Dataset S2, and the results were shown in Fig. 5 (Additional file 1: Tables S7A-D). As shown in Fig. 5a-d, most scoring measures attain their maximum when *L* = 9. Hence, *L* = 9 is selected in the definition of the AGEZ. Meanwhile, using Score8, the model correctly predicts 37 in Additional file 2: Dataset S1 (Fig. 5a) and 57 in Additional file 3: Datasets S2 (Fig. 5b) based on the relative frequencies shown in Tables S4 and S5, respectively (Additional file 1: Tables S7A-B). Similarly, 34 and 55 BPSs were correctly predicted using Additional file 2: Dataset S1 (Fig. 5c) and S2 (Fig. 5d) under the relative frequency shown in Table S6 (Additional file 1: Tables S7C-D). The Score 8 shows the best performance, and is therefore chosen for human BPS prediction.

Taking the BPS prediction on Additional file 2: Dataset S1 as an example, 9 out of 42 introns were incorrectly predicted. In fact, when we compared the scores of all the heptamers in these 9 introns, most of them (8 out of 9 introns) ranked quite high (2nd-4th based on the score value) and they are likely potential BPSs as well. However, as our model adopts a stringent criteria to select only the top-ranking heptamer as the candidate BPS, those heptamers ranked slightly lower were not chosen. In addition, the degeneracy of BPSs in human genome complicates the prediction based on the conservative property of a short sequence alone. The scoring scheme in our model is based on a limited number of experimentally verified human introns, the model is expected to be improved when more reliable data become available. Nonetheless, despite these limitations, our model appeared to be quite efficient in quantifying the splicing strength of putative BPSs based on the results generated.

### Comparison with other prediction models

To evaluate the performance of our model, a comparison with other four previously published models was conducted on Additional file 2: Dataset S1 and Additional file 3: Dataset S2 for BPS prediction, and the corresponding accuracies are shown in Table 1. Here, the accuracy was defined as the number of introns with correctly predicted branch site divided by the total number of introns in the dataset.

**Table 1:** The accuracies (%) of predicting models on Additional file 2: Dataset S1 and Additional file 3: Dataset S2

Methods	Additional file 2: Dataset S1	Additional file 3: Dataset S2
I	88.10	64.77
II	80.96	62.50
III	76.19	53.41
IV	59.52	43.18
V	50.00	38.64
VI	45.24	35.23

Methods I and II are our method based on relative frequencies I and II, respectively. Methods III, IV, V and VI were developed by Corvelo, Gooding, Plass and Schwartz, respectively

Of four methods compared, Plass et al. [18] and Schwartz et al. [19] search over the fixed regions of 100 nts and 200 nts, respectively, and the BPSs are ranked according to the Hamming distance to a strict consensus TACTAAC. Gooding et al. [17] and Corvelo et al. [21] search for candidate BPS in the AGEZ region and score candidates using a PWM trained from human BPS dataset. Corvelo et al. [21] uses SVM model combined with PPT associated features, and so far this method gives the best results for BPS prediction.

From Table 1, our method both achieved the best performance with accuracies of 88.10%/80.95% for Additional file 2: Dataset S1 and 64.77%/62.50% for Additional file 3: Dataset S2, respectively. For SVM model, the accuracies are 76.19% and 53.41% for S1 and S2, respectively. Gooding et al. model gives 59.52% and 43.18% accuracy for S1 and S2 respectively, whereas hamming distance performs the worst due to its high stringency.

### Contributions of the PPT and the BPS-U2 snRNP binding energy

The observation that the BPS appears to be dependent on the presence of consecutive pyrimidines near the 3′ end of the PPT led us to suspect that the PPT may contribute to the splicing strength of BPS signal, and affect the base composition near the 3’ss of intron is the consequence of the branch site position. We dynamically search the branch site within the shortened AGEZ to avoid the influences of other elements in the intron and the prediction accuracy was successfully improved.

To investigate whether the binding energy of BPS-U2 snRNP contributes to the molecular recognition in splicing, a comparison was made between Score0 and Score8 in Fig. 5a as an example. Because all BPSs in Additional file 2: Dataset S1 are in "TN*A*" model, which means the ${f}_{6,{X}_6}$ in Score8 always equals to 1. The difference between Score0 and Score8 is *BE*(*X*/*X*_6_), which is the binding energy between U2 snRNP and the BPS excluding the branch site (*k* = 6). As illustrated in Fig. 5a, Score0 and Score8 both attain the maximum at *L* = 9, and by adding the BPS-U2 snRNP binding energy, the number of correctly predicted introns was increased from 34 to 37. The similar results could be found in Fig. 5b-d. Taken together, these demonstrate that the binding energy of BPS-U2 snRNP can affect the selection of branch site, hence is an important factor contributes to our model’s performance.

### Genome-wide BPS prediction in human introns

**Fig. 6:**
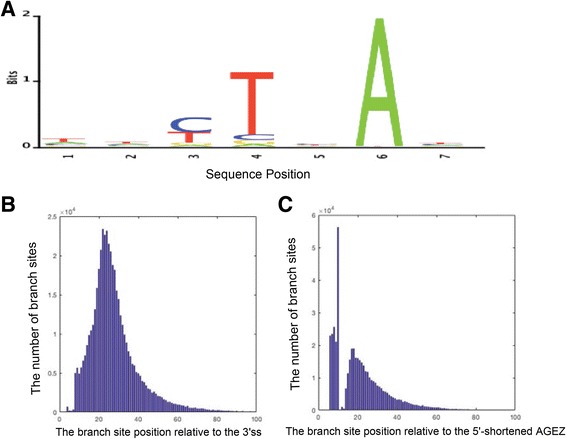
Sequence logo for genome-wide BPSs predicted by our method (**a**), and the branch site positions relative to the 3’ss (**b**) and the 5′-end of the shortened AGEZ (**c**)

Under the relative frequencies of BPSs predicted by sequencing method [24], a genome-wide BPS prediction was carried out on all human introns (>100 bp) with GT as 5’ss and AG as 3’ss [24, 36], and a total of 462,881 BPSs were predicted by our method. The characteristics of genome-wide predicted BPSs were plotted in Fig. 6a (Additional file 1: Table S8). The results suggest that the human consensus BPS should be YTN*A*Y [26], which is in accordance with experimentally verified human BPSs. In addition, the branch site positions relative to the 3’ss and the 5′ end of the shortened AGEZ were illustrated in Fig. 6b-c. As seen, most branch sites appear around the −18 to −32 nts from the 3’ss, and branch sites frequently locate towards the 5′ end of the shortened AGEZ, which are consistent with the conclusions by Corvelo et al. [21], Mercer et al. [24], and Pastuszak et al. [37].

## Conclusions

BPS recognition by the U2 snRNP is an important event in pre-mRNA splicing. However, the characteristics of BPS motif under different splicing states, and the relationships between the BPS element and other splicing factors are largely unknown. In silico prediction of BPS in human introns are challenged by degeneracy of BPS motifs and lacking of experimentally verified BPS datasets. In this paper, we develop a simple yet efficient heuristic model for BPS prediction in a dynamic search region based on a new statistical measurement. This newly defined BPS search region can effectively avoid the influences of other elements in the intron and increase the BPS prediction accuracy. The binding energy of BPS-U2 snRNP and nucleotide preference of branch site were taken into consideration when the splicing strength of putative BPS is measured. We show that our method gave the best performance when compared with other current prediction methods. The improved performance indicates that the binding energy of BPS-U2 snRNP contributes to the molecular recognition during the pre-mRNA splicing process. In addition, a genome-wide human BPS prediction was carried out based on our model. The characteristics of predicted BPSs are in accordance with experimentally verified human BPSs, and branch site positions relative to the 3’ss and the 5’end of the shortened AGEZ are consistent with the results of published papers.

Although BPS predictions based on sequence consensus have achieved certain successes, most predicted BPSs have not been experimentally verified. This makes it difficult to evaluate the relationship between statistical prediction and biological success of the models. Moreover, efforts to computationally identify BPS are challenged by the variable locations of BPSs within the intron. Further improvement of the software relies on the availability of more experimentally validated human BPSs. Our current work mainly focus on human BPS prediction. Hopefully, we could extend BPS prediction to other species in the further work.

## Additional files

Additional file 1: Table S1. The formulas of 15 scoring measures (Score0-Score14) and corresponding values for *P*_*j*_, Q_*j*_ ∈ [0, 1], *j* = 1, 2, 3. **Table S2**. The endpoints (5′- and 3′-) of the shortened AGEZ and corresponding branch sites labeled by their positons relative to the 3’ss for each intron in Additional file 2: Dataset S1 when *L* = 9. **Table S3**. The endpoints (5′- and 3′-) of the shortened AGEZ and corresponding branch sites labeled by their positons relative to the 3’ss for each intron in Additional file 3: Dataset S2 when *L* = 9. **Table S4**. The relative frequencies of nucleotides at each position for BPSs in Additional file 2: Dataset S1 and corresponding information content (IC). **Table S5**. The relative frequencies of nucleotides at each position for BPSs in Additional file 3: Dataset S2 and corresponding information content (IC). **Table S6**. The relative frequencies of nucleotides at each position for 252,302 human BPSs predicted by sequencing method. **Table S7.** The results of 15 scoring measures (Score0-Score14) for BPS prediction on Additional file 2:Dataset S1 and Additional file 3: Dataset S2. **Table S8**. The relative frequencies of nucleotides at each position for genome-wide predicted BPSs and corresponding information content (IC) (DOCX 44 kb)

Additional file 2: Dataset_S1. (FASTA 146 kb)

Additional file 3: Dataset_S2. (FASTA 251 kb)

## Abbreviations

3’ss: the 3′-splice site
5’ss: the 5′-splice site
AGEZ: the AG dinucleotide exclusion zone
BPS: Branch point sequence
PPT: the polypyrimidine tract
PSSM: Position-specific scoring matrix
SF1: the splicing factor 1
SVM: Support Vector Machine
U2AF35: the 35 kDa subunit of U2AF
U2AF65: the 65 kDa subunit of U2AF
