# Supplementary material for: A heuristic model for computational prediction of human branch point sequence
Source: BMC Bioinformatics. 2017 Oct 24;18:459. doi: 10.1186/s12859-017-1864-9 (PMC5655975; doi:10.1186/s12859-017-1864-9)
Supplement: Supplementary file 1 — The formulas of 15 scoring measures (Score0-Score14) and corresponding values for P j, Qj ∈ [0, 1], j = 1, 2, 3. Table S2. The endpoints (5′- and 3′-) of the shortened AGEZ and corresponding branch sites labeled by their positons relative to the 3’ss for each intron in Additional file 2: Dataset S1 when L = 9. Table S3. The endpoints (5′- and 3′-) of the shortened AGEZ and corresponding branch sites labeled by their positons relative to the 3’ss for each intron in Additional file 3: Dataset S2 when L = 9. Table S4. The relative frequencies of nucleotides at each position for BPSs in Additional file 2: Dataset S1 and corresponding information content (IC). Table S5. The relative frequencies of nucleotides at each position for BPSs in Additional file 3: Dataset S2 and corresponding information content (IC). Table S6. The relative frequencies of nucleotides at each position for 252,302 human BPSs predicted by sequencing method. Table S7. The results of 15 scoring measures (Score0-Score14) for BPS prediction on Additional file 2:Dataset S1 and Additional file 3: Dataset S2. Table S8. The relative frequencies of nucleotides at each position for genome-wide predicted BPSs and corresponding information content (IC) (DOCX 44 kb) [file 12859_2017_1864_MOESM1_ESM.docx]

**A heuristic model for** **computational prediction of human branch point sequence**

Jia Wen, Jue Wang, Qing Zhang and Dianjing Guo

School of Life Science and State Key Laboratory of Agrobiotechnology, The Chinese University of Hong Kong, Hong Kong, China.

**Table S1**. The formulas of 15 scoring measures (Score0-Score14) and corresponding values for $P_{j}, Q_{j}\in\left[ 0,1 \right], j=1,2,3$.

| Scoring measures | Formula and corresponding values for $P_{j}, Q_{j}\in\left[ 0,1 \right], j=1,2,3$.* |
| --- | --- |
| Score0 | $\sum_{i=1}^{7} {log}_{2}\left( f_{i,X_{i}} \right)$, $P_{j}=0, Q_{j}=0, j=1,2,3$. |
| Score1 | $\sum_{i=1}^{7} {log}_{2}\left( f_{i,X_{i}} \right)-BE(X/X_{6})$, $P_{2}=1$. |
| Score2 | $\sum_{i=1}^{7} {log}_{2}\left( f_{i,X_{i}} \right)-BE(X/X_{5})$, $P_{1}=1$. |
| Score3 | $\sum_{i=1}^{7} {log}_{2}\left( f_{i,X_{i}} \right)-BE(X/X_{7})$, $P_{3}=1$. |
| Score4 | $\sum_{i=1}^{7} {log}_{2}\left( f_{i,X_{i}} \right)-(BE(X/X_{6})+BE(X/X_{5}))/2$, $P_{1}, P_{2}=1$. |
| Score5 | $\sum_{i=1}^{7} {log}_{2}\left( f_{i,X_{i}} \right)-(BE(X/X_{6})+BE(X/X_{7}))/2$, $P_{2}, P_{3}=1$. |
| Score6 | $\sum_{i=1}^{7} {log}_{2}\left( f_{i,X_{i}} \right)-(BE(X/X_{5})+BE(X/X_{7}))/2$, $P_{1}, P_{3}=1$. |
| Score7 | $\sum_{i=1}^{7} {log}_{2}\left( f_{i,X_{i}} \right)-(BE(X/X_{6})+BE(X/X_{5})+BE(X/X_{7}))/3$, $P_{1}, P_{2}, P_{3}=1$. |
| Score8 | $\sum_{i=1}^{7} {log}_{2}\left( f_{i,X_{i}} \right)-BE(X/X_{6})*f_{6,X_{6}}$, $P_{2}=1, Q_{2}=1$. |
| Score9 | $\sum_{i=1}^{7} {log}_{2}\left( f_{i,X_{i}} \right)-BE(X/X_{5})*f_{5,X_{5}}$, $P_{1}=1, Q_{1}=1$. |
| Score10 | $\sum_{i=1}^{7} {log}_{2}\left( f_{i,X_{i}} \right)-BE(X/X_{7})*f_{7,X_{7}}$, $P_{3}=1, Q_{3}=1$. |
| Score11 | $\sum_{i=1}^{7} {log}_{2}\left( f_{i,X_{i}} \right)-(BE(X/X_{6})*f_{6,X_{6}}+{BE(X/X_{5})f}_{5,X_{5}})/2$, $P_{1}, P_{2}=1, {Q_{1},Q}_{2}=1$. |
| Score12 | $\sum_{i=1}^{7} {log}_{2}\left( f_{i,X_{i}} \right)-(BE(X/X_{6})*f_{6,X_{6}}+{BE(X/X_{7})f}_{7,X_{7}})/2$, $P_{2}, P_{3}=1, {Q_{2},Q}_{3}=1$. |
| Score13 | $\sum_{i=1}^{7} {log}_{2}\left( f_{i,X_{i}} \right)-(BE(X/X_{5})*f_{5,X_{5}}+{BE(X/X_{7})f}_{7,X_{7}})/2$, $P_{1}, P_{3}=1, {Q_{1},Q}_{3}=1$. |
| Score14 | $\sum_{i=1}^{7} {log}_{2}\left( f_{i,X_{i}} \right)-(BE(X/X_{5})*f_{5,X_{5}}+{BE(X/X_{6})f}_{6,X_{6}}+{BE(X/X_{7})f}_{7,X_{7}})/3$, $P_{j}=1,Q_{j} =1, j=1,2,3$. |

*The unstated parameters for $P_{j}, Q_{j}, j=1,2,3$ equal to 0.

**Table S2**. The endpoints (5’- and 3’-) of the shortened AGEZ and corresponding branch sites labeled by their positons relative to the 3’ss for each intron in dataset S1 when *L*=9.

| No. | Intron | Branch site | 3’-shortened AGEZ | 5’-shortened AGEZ |
| --- | --- | --- | --- | --- |
| 1 | INT[chr11:122437248-122437981:-] | 23 | 16 | 28 |
| 2 | INT[chr11:14945977-14948056:-] | 36 | 31 | 43 |
| 3 | INT[chr1:149639946-149640338:+] | 40, 44 | 9 | 49 |
| 4 | INT[chr11:5204606-5204735:-] | 37 | 7 | 54 |
| 5 | INT[chr11:5227397-5227518:-] | 30 | 18 | 59 |
| 6 | INT[chr11:5247483-5247604:-] | 31 | 8 | 58 |
| 7 | INT[chr1:154547611-154548455:-] | 24 | 10 | 45 |
| 8 | INT[chr1:154570059-154572241:-] | 21 | 9 | 27 |
| 9 | INT[chr12:21691218-21698743:-] | 22 | 7 | 40 |
| 10 | INT[chr15:70288287-70289067:-] | 16 | 15 | 76 |
| 11 | INT[chr15:70289255-70289741:-] | 16 | 7 | 42 |
| 12 | INT[chr15:70289874-70296803:-] | 21 | 20 | 42 |
| 13 | INT[chr15:70296896-70298338:-] | 31 | 13 | 76 |
| 14 | INT[chr16:163007-163123:+] | 19 | 13 | 74 |
| 15 | INT[chr16:163329-163470:+] | 18 | 17 | 57 |
| 16 | INT[chr16:29986484-29987467:+] | 25 | 10 | 53 |
| 17 | INT[chr16:88155661-88156243:+] | 22 | 20 | 50 |
| 18 | INT[chr17:16225224-16225940:+] | 28 | 12 | 42 |
| 19 | INT[chr1:8849897-8850633:-] | 27 | 16 | 48 |
| 20 | INT[chr1:8850704-8853097:-] | 21 | 20 | 47 |
| 21 | INT[chr19:757557-758868:+] | 351 | 9 | 391 |
| 22 | INT[chr20:2391874-2392392:-] | 25 | 9 | 59 |
| 23 | INT[chr20:2394466-2396252:-] | 34 | 11 | 65 |
| 24 | INT[chr20:2396405-2399333:-] | 24 | 13 | 39 |
| 25 | INT[chr20:3708423-3709825:-] | 160, 166 | 16 | 196 |
| 26 | INT[chr22:38040160-38040636:-] | 21 | 18 | 36 |
| 27 | INT[chr22:38043581-38044350:-] | 21 | 14 | 36 |
| 28 | INT[chr22:40379641-40382852:+] | 26 | 14 | 33 |
| 29 | INT[chr3:14184885-14186941:-] | 24 | 11 | 62 |
| 30 | INT[chr5:147825682-147869211:-] | 86 | 20 | 112 |
| 31 | INT[chr5:147869781-147882948:-] | 213 | 7 | 232 |
| 32 | INT[chr5:147883103-147908423:-] | 267 | 12 | 302 |
| 33 | INT[chr5:147908625-147909892:-] | 143, 151 | 142 | 160 |
| 34 | INT[chr6:29679473-29680249:-] | 275 | 13 | 298 |
| 35 | INT[chr6:44322911-44324344:+] | 18 | 12 | 24 |
| 36 | INT[chr6:44327983-44328759:+] | 22 | 19 | 31 |
| 37 | INT[chr6:74285961-74286326:-] | 28 | 9 | 33 |
| 38 | INT[chr6:74286501-74287443:-] | 23 | 19 | 30 |
| 39 | INT[chrX:77246559-77252019:+] | 28 | 14 | 42 |
| 40 | INT[chrX:77256314-77259464:+] | 29 | 13 | 45 |
| 41 | INT[chrX:77259569-77260203:+] | 21 | 10 | 38 |
| 42 | INT[chrX:77265528-77267026:+] | 27 | 8 | 41 |

**Table S3**. The endpoints (5’- and 3’-) of the shortened AGEZ and corresponding branch sites labeled by their positons relative to the 3’ss for each intron in dataset S2 when *L*=9.

| No. | Intron | Branch site | 3’-shortened AGEZ | 5’-shortened AGEZ |
| --- | --- | --- | --- | --- |
| 1 | CALCA4 | 36 | 31 | 43 |
| 2 | CALCA3 | 23, 24, 32 | 12 | 53 |
| 3 | CSH11 | 23 | 11 | 63 |
| 4 | DQB13 | 21 | 8 | 24 |
| 5 | GH11 | 22, 28, 36 | 11 | 63 |
| 6 | GH14 | 24, 37, 38 | 28 | 46 |
| 7 | HBB1 | 37 | 7 | 54 |
| 8 | HBE11 | 31 | 8 | 58 |
| 9 | HBG11 | 30 | 18 | 59 |
| 10 | MYH105 | 31 | 14 | 56 |
| 11 | XPC3 | 24 | 11 | 62 |
| 12 | ACTB3 | 30 | 14 | 39 |
| 13 | CCT34 | 21 | 9 | 27 |
| 14 | CCT311 | 24, 10 | 10 | 45 |
| 15 | EEF1A12 | 28, 19 | 9 | 33 |
| 16 | EEF1A11 | 23 | 19 | 30 |
| 17 | ENO12 | 27, 25 | 20 | 77 |
| 18 | ENO14 | 21 | 20 | 47 |
| 19 | ENO15 | 27, 24 | 16 | 48 |
| 20 | ENO16 | 30, 27, 26 | 10 | 41 |
| 21 | ENO17 | 38, 28, 27, 26 | 11 | 63 |
| 22 | ENO111 | 48, 47, 46 | 17 | 62 |
| 23 | G22P11 | 30, 28 | 15 | 44 |
| 24 | G22P18 | 31, 26, 25 | 14 | 33 |
| 25 | G22P110 | 33 | 8 | 39 |
| 26 | HSPA81 | 23, 19, 17 | 16 | 28 |
| 27 | HSPCB6 | 22, 23 | 10 | 53 |
| 28 | HSPCB10 | 24, 22 | 19 | 31 |
| 29 | HSPCB1 | 18 | 12 | 24 |
| 30 | LDHB2 | 24, 23, 22 | 7 | 40 |
| 31 | PGK11 | 29, 28 | 14 | 42 |
| 32 | PGK12 | 26 | 17 | 48 |
| 33 | PGK14 | 33, 29 | 13 | 45 |
| 34 | PGK15 | 29, 22, 21 | 10 | 38 |
| 35 | PGK16 | 28, 23 | 13 | 36 |
| 36 | PGK18 | 27 | 8 | 41 |
| 37 | PGK110 | 36 | 15 | 48 |
| 38 | PKM22 | 31, 29, 25, 23 | 13 | 76 |
| 39 | PKM23 | 25, 21 | 20 | 42 |
| 40 | PKM24 | 38, 33, 23, 16, 8 | 7 | 42 |
| 41 | PKM25 | 32, 31, 28, 20, 18, 16 | 15 | 76 |
| 42 | PKM26 | 39, 34, 32 | 28 | 86 |
| 43 | PKM28 | 65, 52, 50 | 9 | 55 |
| 44 | PKM210 | 27, 26, 25 | 20 | 66 |
| 45 | PSMB44 | 44, 40 | 9 | 49 |
| 46 | RPL134 | 26, 23, 22 | 20 | 50 |
| 47 | RPL135 | 22, 20 | 14 | 83 |
| 48 | RPL13A1 | 22 | 10 | 35 |
| 49 | RPL32 | 21, 19 | 14 | 36 |
| 50 | RPL34 | 24 | 11 | 31 |
| 51 | RPL35 | 29 | 10 | 30 |
| 52 | RPL36 | 22, 21 | 18 | 36 |
| 53 | RPL84 | 23 | 10 | 29 |
| 54 | SLC25A32 | 31 | 16 | 51 |
| 55 | SNRPB1 | 24 | 13 | 39 |
| 56 | SNRPB2 | 36, 35, 34 | 11 | 65 |
| 57 | SNRPB3 | 30, 29, 28, 27 | 8 | 31 |
| 58 | SNRPB4 | 27, 26, 25 | 9 | 59 |
| 59 | SNRPB6 | 27 | 12 | 42 |
| 60 | UBB1 | 30, 29, 28 | 12 | 42 |
| 61 | COL5A132 | 27 | 18 | 29 |
| 62 | FBN230 | 16 | 8 | 33 |
| 63 | GH13 | 21 | 14 | 35 |
| 64 | ITGB431 | 17 | 16 | 39 |
| 65 | LCAT4 | 20 | 19 | 39 |
| 66 | LDLR9 | 25 | 22 | 50 |
| 67 | NPC16 | 28 | 8 | 46 |
| 68 | PMM27 | 23 | 9 | 39 |
| 69 | TH11 | 22 | 11 | 47 |
| 70 | TSC238 | 18 | 3 | 45 |
| 71 | XRCC69 | 26 | 14 | 33 |
| 72 | MBNL16 | 141, 144 | 13 | 184 |
| 73 | MBNL18 | 51, 64 | 28 | 145 |
| 74 | MBNL19 | 31, 41, 229 | 15 | 235 |
| 75 | CLK14 | 224, 229 | 18 | 246 |
| 76 | CLK34 | 196 | 48 | 218 |
| 77 | HTR43 | 143, 151 | 142 | 160 |
| 78 | HTR44 | 27, 33, 72, 267, 273 | 12 | 302 |
| 79 | HTR45 | 26, 39, 213 | 7 | 232 |
| 80 | HTR4g | 31, 86 | 20 | 112 |
| 81 | HBA21 | 19 | 13 | 74 |
| 82 | HBA22 | 18 | 17 | 57 |
| 83 | ALDOA8 | 25 | 10 | 53 |
| 84 | PTBP110 | 351 | 9 | 391 |
| 85 | SPEF11 | 160, 166 | 16 | 196 |
| 86 | GABBR117 | 275 | 13 | 298 |
| 87 | HSP90AB11 | 18 | 12 | 24 |
| 88 | HSP90AB110 | 22 | 19 | 31 |

**Table S4**. The relative frequencies of nucleotides at each position for BPSs in dataset S1 and corresponding information content (IC).

| Nucleotide | Posit. 1 | Posit. 2 | Posit. 3 | Posit. 4 | Posit. 5 | Posit. 6 | Posit. 7 |
| --- | --- | --- | --- | --- | --- | --- | --- |
| A | 0.244 | 0.400 | 0.067 | 0 | 0.289 | 1.000 | 0.133 |
| C | 0.289 | 0.156 | 0.600 | 0 | 0.311 | 0 | 0.422 |
| G | 0.133 | 0.133 | 0.111 | 0 | 0.267 | 0 | 0.067 |
| T | 0.333 | 0.311 | 0.222 | 1.000 | 0.133 | 0 | 0.378 |
| IC | 0.07 | 0.14 | 0.46 | 2.00 | 0.06 | 2.00 | 0.30 |

**Table S5**. The relative frequencies of nucleotides at each position for BPSs in dataset S2 and corresponding information content (IC).

| Nucleotide | Posit. 1 | Posit. 2 | Posit. 3 | Posit. 4 | Posit. 5 | Posit. 6 | Posit. 7 |
| --- | --- | --- | --- | --- | --- | --- | --- |
| A | 0.231 | 0.249 | 0.127 | 0.110 | 0.162 | 0.676 | 0.231 |
| C | 0.237 | 0.214 | 0.422 | 0.191 | 0.376 | 0.098 | 0.306 |
| G | 0.214 | 0.237 | 0.145 | 0.092 | 0.225 | 0.064 | 0.139 |
| T | 0.318 | 0.301 | 0.306 | 0.607 | 0.237 | 0.162 | 0.324 |
| IC | 0.02 | 0.01 | 0.17 | 0.44 | 0.07 | 0.61 | 0.07 |

**Table S6**. The relative frequencies of nucleotides at each position for 252302 human BPSs predicted by sequencing method.

| Nucleotide | Posit. 1 | Posit. 2 | Posit. 3 | Posit. 4 | Posit. 5 | Posit. 6 | Posit. 7 |
| --- | --- | --- | --- | --- | --- | --- | --- |
| A | 58724/  0.2328 | 62824/  0.2490 | 45150/  0.1790 | 31571/  0.1251 | 48763/  0.1933 | 192826/  0.7643 | 68503/  0.2715 |
| C | 53913/  0.2137 | 56545/  0.2241 | 81708/  0.3238 | 47467/  0.1881 | 74961/  0.2971 | 23338/  0.0925 | 72358/  0.2868 |
| G | 54941/  0.2178 | 49289/  0.1954 | 44798/  0.1776 | 34086/  0.1351 | 53231/  0.2110 | 10749/  0.0426 | 43790/  0.1736 |
| T | 84724/  0.3358 | 83644/  0.3315 | 80646/  0.3196 | 139178/  0.5516 | 75347/  0.2986 | 25389/  0.1006 | 67651/  0.2681 |

**Table S7.** The results of 15 scoring measures (Score0-Score14) for BPS prediction on datasets S1 and S2.

| 1. Dataset S1 with relative frequencies shown in Table S4. | | | | | | |
| --- | --- | --- | --- | --- | --- | --- |
| Scoring measure | L=7 | L=8 | L=9 | L=10 | L=11 | L=12 |
| Score0 | 31 | 33 | 34 | 34 | 34 | 33 |
| Score1 | 33 | 35 | 37 | 37 | 37 | 36 |
| Score2 | 31 | 33 | 35 | 35 | 35 | 34 |
| Score3 | 33 | 35 | 37 | 37 | 37 | 36 |
| Score4 | 32 | 34 | 36 | 36 | 36 | 35 |
| Score5 | 33 | 35 | 37 | 37 | 37 | 36 |
| Score6 | 32 | 34 | 36 | 36 | 36 | 35 |
| Score7 | 33 | 35 | 37 | 37 | 37 | 36 |
| Score8 | 33 | 35 | 37 | 37 | 37 | 36 |
| Score9 | 32 | 34 | 35 | 35 | 35 | 34 |
| Score10 | 32 | 34 | 35 | 35 | 35 | 34 |
| Score11 | 32 | 34 | 36 | 36 | 36 | 35 |
| Score12 | 32 | 34 | 36 | 36 | 36 | 35 |
| Score13 | 32 | 34 | 35 | 35 | 35 | 34 |
| Score14 | 32 | 34 | 36 | 36 | 36 | 35 |
| 1. Dataset S2 with relative frequencies shown in Table S5. | | | | | | |
| Scoring measure | L=7 | L=8 | L=9 | L=10 | L=11 | L=12 |
| Score0 | 44 | 48 | 49 | 48 | 45 | 45 |
| Score1 | 45 | 49 | 50 | 49 | 47 | 45 |
| Score2 | 45 | 48 | 49 | 48 | 46 | 45 |
| Score3 | 43 | 47 | 48 | 47 | 45 | 44 |
| Score4 | 47 | 50 | 51 | 50 | 48 | 47 |
| Score5 | 45 | 49 | 50 | 49 | 47 | 45 |
| Score6 | 48 | 51 | 52 | 51 | 49 | 48 |
| Score7 | 49 | 52 | 53 | 52 | 50 | 49 |
| Score8 | 52 | 56 | 57 | 56 | 54 | 52 |
| Score9 | 44 | 48 | 49 | 48 | 46 | 45 |
| Score10 | 47 | 51 | 52 | 51 | 48 | 48 |
| Score11 | 48 | 52 | 53 | 52 | 50 | 49 |
| Score12 | 49 | 53 | 54 | 53 | 50 | 49 |
| Score13 | 45 | 49 | 50 | 49 | 47 | 46 |
| Score14 | 48 | 52 | 53 | 52 | 50 | 49 |
| 1. Dataset S1 with relative frequencies shown in Table S6. | | | | | | |
| Scoring measure | L=7 | L=8 | L=9 | L=10 | L=11 | L=12 |
| Score0 | 23 | 25 | 25 | 24 | 22 | 22 |
| Score1 | 31 | 32 | 33 | 32 | 31 | 30 |
| Score2 | 28 | 29 | 30 | 29 | 28 | 28 |
| Score3 | 26 | 28 | 28 | 27 | 26 | 26 |
| Score4 | 32 | 33 | 34 | 33 | 32 | 32 |
| Score5 | 30 | 31 | 31 | 30 | 29 | 28 |
| Score6 | 30 | 31 | 31 | 30 | 29 | 28 |
| Score7 | 31 | 32 | 33 | 32 | 31 | 31 |
| Score8 | 32 | 33 | 34 | 33 | 31 | 30 |
| Score9 | 24 | 26 | 26 | 25 | 24 | 24 |
| Score10 | 23 | 25 | 25 | 24 | 22 | 22 |
| Score11 | 30 | 31 | 31 | 30 | 29 | 29 |
| Score12 | 30 | 31 | 31 | 30 | 28 | 28 |
| Score13 | 23 | 25 | 25 | 24 | 22 | 22 |
| Score14 | 29 | 30 | 30 | 29 | 27 | 27 |
| 1. Dataset S2 with relative frequencies shown in Table S6. | | | | | | |
| Scoring measure | L=7 | L=8 | L=9 | L=10 | L=11 | L=12 |
| Score0 | 40 | 42 | 41 | 40 | 37 | 37 |
| Score1 | 47 | 49 | 51 | 50 | 48 | 47 |
| Score2 | 43 | 45 | 47 | 46 | 44 | 44 |
| Score3 | 42 | 45 | 45 | 44 | 42 | 42 |
| Score4 | 48 | 50 | 52 | 51 | 49 | 49 |
| Score5 | 46 | 48 | 48 | 47 | 45 | 44 |
| Score6 | 47 | 49 | 49 | 48 | 46 | 46 |
| Score7 | 48 | 50 | 52 | 51 | 49 | 49 |
| Score8 | 51 | 53 | 55 | 54 | 51 | 50 |
| Score9 | 40 | 43 | 43 | 42 | 40 | 40 |
| Score10 | 42 | 43 | 43 | 42 | 40 | 40 |
| Score11 | 49 | 51 | 51 | 50 | 48 | 48 |
| Score12 | 50 | 51 | 51 | 50 | 47 | 47 |
| Score13 | 41 | 44 | 44 | 43 | 40 | 40 |
| Score14 | 47 | 49 | 49 | 48 | 45 | 45 |

**Table S8**. The relative frequencies of nucleotides at each position for genome-wide predicted BPSs and corresponding information content (IC).

| Nucleotide | Posit. 1 | Posit. 2 | Posit. 3 | Posit. 4 | Posit. 5 | Posit. 6 | Posit. 7 |
| --- | --- | --- | --- | --- | --- | --- | --- |
| A | 99562/  0.2151 | 113185/  0.2445 | 25409/  0.0549 | 16254/  0.0351 | 77484/  0.1674 | 461291/  0.9966 | 106829/  0.2308 |
| C | 83428/  0.1802 | 96373/  0.2082 | 228746/  0.4942 | 31615/  0.0683 | 153871/  0.3324 | 464/  0.0010 | 143398/  0.3098 |
| G | 79896/  0.1726 | 77233/  0.1669 | 38429/  0.0830 | 24856/  0.0537 | 112771/  0.2436 | 10/  0.0000 | 68129/  0.1472 |
| T | 199995/  0.4321 | 176090/  0.3804 | 170297/  0.3679 | 390156/  0.8429 | 118755/  0.2566 | 1116/  0.0024 | 144525/  0.3122 |
| IC | 0.1170 | 0.0703 | 0.4388 | 1.1305 | 0.0403 | 1.9638 | 0.0569 |
